# Mutant-Dependent Local Orientational Correlation in Biofilms of *Vibrio campbellii* Revealed through Digital Processing of Light Microscopy Images

**DOI:** 10.3390/ijms24065423

**Published:** 2023-03-12

**Authors:** Maura Cesaria, Matteo Calcagnile, Pietro Alifano, Rosella Cataldo

**Affiliations:** 1 Department of Mathematics and Physics Ennio De Giorgi, University of Salento-c/o Campus Ecotekne, Via per Arnesano, 73100 Lecce, Italy; 2 Department of Biological and Environmental Sciences and Technologies (Di.S.Te.BA.), University of Salento-c/o Campus Ecotekne—S.P. 6, 73100 Lecce, Italy

**Keywords:** *Vibrio campbellii* strains, high-coverage mature biofilms, light microscopy imaging, digital image processing, correlation statistics, mutant-dependent oriented subdomains

## Abstract

Biofilms are key bacterial communities in genetic and adaptive resistance to antibiotics as well as disease control strategies. The mature high-coverage biofilm formations of the *Vibrio campbellii* strains (wild type BB120 and isogenic derivatives JAF633, KM387, and JMH603) are studied here through the unstraightforward digital processing of morphologically complex images without segmentation or the unrealistic simplifications used to artificially simulate low-density formations. The main results concern the specific mutant- and coverage-dependent short-range orientational correlation as well as the coherent development of biofilm growth pathways over the subdomains of the image. These findings are demonstrated to be unthinkable based only on a visual inspection of the samples or on methods such as Voronoi tessellation or correlation analyses. The presented approach is general, relies on measured rather than simulated low-density formations, and could be employed in the development of a highly efficient screening method for drugs or innovative materials.

## 1. Introduction

Biofilms are organized surface-bound communities of bacterial cells and aggregates (bacteria and/or fungi) embedded in a three-dimensional self-produced matrix consisting of hydrated polymeric substances, polysaccharides, nucleic acids, proteins, lipids, and other components that house bacteria and act as a physical barrier to protect them against environmental conditions and attacks [1,2,3].

Compared to the planktonic state, pathogenic strains within biofilms not only differ in their organization and phenotypes but also exhibit enhanced virulence and resistance to antibacterial agents because of the effective resilience of biofilms to external conditions. In addition, biofilms are also associated with adaptive and transient resistance manifested through the acquisition of the “persistent” phenotype: a bacterial subpopulation that is not sensitive to the action of an antibiotic but tolerates it [4].

Biofilms are ubiquitous in natural, industrial, and clinical habitats/environments and are difficult to eradicate. They are a primary cause of persistent infections with a very difficult clinical treatment due to the high tolerance and resistance of the bacteria to antimicrobial treatments and agents and the subsequent severe infections hamper the recovery process, especially in patients with already weakened immune systems [5,6].

In general, not only is the treatment of device-associated and device-unassociated infections is critical, but, above all, it remains incompletely understood how these infections develop, whether an infection is only caused by a selection of strains, and whether adaptation increases biofilm formation. Additionally, if so, what is the role played by each element concurring in adaptation [7]? Biofilm development progresses from initial planktonic bacterial mass transport towards a surface to several steps, including bacterial mass transport towards a surface, initial reversible bacterial adhesion, a transition to irreversible attachment to a biocompatible surface, the formation of microcolonies that grow and divide, the secretion of the extracellular matrix and biofilm formation, dispersion for further colonization, and the optimization of the available resources.

Numerous environmental factors affect this evolution, such as lapsing time, moisture, pH, temperature, the availability of nutrients, the presence of oxygen, the characteristics of the abiotic and biotic attachment surfaces (surface energy, charge, topography, and substratum stiffness on adhesion), biofilm–surface interactions, the hydrodynamics of aqueous media, the water content, available nutrients, and strain mutants [8,9]. In a recent paper, the bacterial contamination of *Staphylococcus aureus ATCC 6538*, *Escherichia coli ATCC 8739*, *Enterococcus sp. ATCC 19952*, *Salmonella enterica subsp*. *enterica serovar Typhi ATCC 6539*, and *Listeria monocytogenes ATCC 7644* on a banknote was studied. The results show both a strong survival rate and low adherence clearly influenced by the material used for the banknotes [10].

Interestingly, growing bacterial biofilms may exhibit a variety of surface morphologies that can reflect their physiological status and nutrient access [11] as well as specific structural genes (flagella, pili, and exopolysaccharide biosynthesis), quorum sensing (QS) regulatory processes [12], self-patterning into microdomains stemming from cell alignment [11,13,14,15,16], and the properties of the matrix in wild-type and mutant strains [17,18,19,20]. In particular, QS is a social behavior that is closely related to cell–cell communication mechanisms and that is activated above a threshold density of the population in which individual cells synthesize and release chemical signaling molecules, termed autoinducers (AIs), which interact with specific receptors and globally respond to them [21]. Multiple AIs may occur and interact with specific receptors. Hence, QS acts as a regulatory mechanism of gene expression triggered by changes in the cell population density that increases the concentration of AIs.

Quorum sensing systems have been identified in both Gram-negative and Gram-positive species of bacteria, and they play a primary role in regulating diverse functions, such as bioluminescence, conjugation, virulence, biofilm formation, and antibiotic production [22]. Recently, inter-*Vibrio*-species quorum sensing inhibition has been investigated by screening bacteria isolated from soil and mud samples in Vietnam. The study discovered a new strain (named XTS1.2.9) that is able to reduce more than 75% of bioluminescence, and these findings were confirmed by testing XTS1.2.9 against different mutants lacking of different quorum sensing AIs. Thus, an intra-genus quorum sensing inhibition mechanism involving CAI-1 was hypothesized to explain such interactions, leading to the conclusion that other investigations on QS are necessary to better understand its scientific aspects and applicative implications [23].

**Figure 1:**
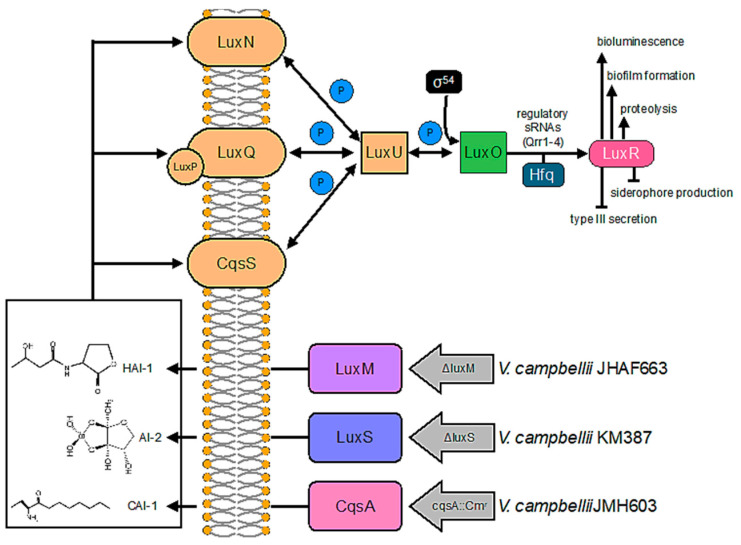
(Color online) The quorum sensing circuit in *V. campbellii* strain BB120 (wild type) and isogenic derivatives JAF633 (Δ*luxM* linked to KanR), KM387 (Δ*luxS*), and JMH603 (*cqsA::Cmr*). All AIs (HAI-1, AI-2, and CAI-1) are active in the case of the wild-type BB120 strain. JAF633 lacks the *LuxM* gene that is involved in the production of HAI-1, KM387 lacks the *LuxS* gene that is involved in the production of AI-2, and JMH603 lacks the *csqA* genes involved in the production of CAI-1. See the text for more information. Figure reworked from Ref. [31].

Quorum sensing has been widely investigated in marine *Vibrio* species due to the easily observable bioluminescence and biofilm formation phenomena [24]. *Vibrio harveyi* ATCC-BAA 1116, reclassified as *V. campbellii* [25], is a marine free-living Gram-negative microorganism which can be found on the surface of algae or as a pathogen in shrimp or fish [26]. *Vibrio campbelli* has a three-channel QS system (as sketched in Figure 1) with three different kinds of AIs that are able to channel information into one phosphorelay cascade [22,24,27]. The first signaling molecule is AI 1 (HAI-1), an acyl-homoserine lactone (N-(3-hydroxybutyryl)-homoserine lactone), which is a specific signaling molecule for *V. harveyi* produced by the synthase *LuxM* [28]. The second AI, that is, AI-2 ((2S, 4S)-2-methyl-2,3,4-tetrahydroxytetrahydrofuran-borate), is a global signaling molecule produced by various bacterial species that is synthesized by the AI synthase *LuxS* [29]. The third AI of the *Vibrio campbellii* QS loop is AI 1 (CAI-1 ((S)-3-hydroxytridecan-4-one)), which is produced by the AI synthase *CqsA* and is specific to the members of the *Vibrio* genus [30]. The three signaling molecules of *Vibrio campbellii* follow a distinct synthesis pattern, and their concentration changes during the growth phase [31]. While the concentration of AI-2 increases during the exponential growth phase, HAI-1 and CAI-1 can be detected only during the late exponential phase. In the QS system of the wild-type BB120 strain, HAI-1, AI-2, and CAI-1 are perceived by three different membrane-bound histidine kinase receptors; that is, *LuxN* binds to HAI-1, *LuxPQ* detects AI-2, and *CqsS* binds to CAI-1 [24].

In this study, we investigated the interplay between QS regulation and biofilm production of four *V. campbellii* strains, that is, the wild-type strain BB120 (also known as ATCC BAA-1116) and the isogenic derivatives JAF633 (Δ*luxM*), KM387 (Δ*luxS*), and JMH603 (*CqsA::Cmr*), which were grown on a hydroxyapatite (HA) substrate. The quantification measurements of the biomass obtained through crystal violet (CV) staining and colony forming unit (CFU) counts were considered together with the deep statistical investigation of the mature biofilm formations imaged through stereomicroscopy.

The proper digital processing and statistical analysis of the images by means of the free-license software Fiji-ImageJ [32,33] and its OrientationJ plugin [34] allowed us to disclose the presence of preferred orientational directions over the subdomains of the images, leading to the conclusion that a mutation-dependent orientational ordering during biofilm development can be highlighted. Differential growth in the *Vibrio cholerae* biofilms, consisting of a mosaic-like distribution of the microdomains stemming from cell verticalization and radial alignment phenomena, was already reported in the literature [11,14,15]. In that case, the information resulted from simulations and the continuous modelling of bacterial samples consisting of a single founder cell to thousands of cells. The cell-to-colony dynamic shaping the structural evolution of the biofilms was investigated through high resolution imaging and computationally demanding simulations, by using thresholding and segmentation of single bacteria arranged in small clusters. A prominent limitation in such an approach is that it may not work for studying realistic biofilms with complex morphologies that are distributed over large areas and are prohibitive to being modelled. Differently, we considered mature biofilm formations, and our orientational analysis was performed on the measured light microscopy images rather than simulated or artificially generated low-density biofilms under simplified conditions. A general workflow was presented for studying, at both the local and global scale, complex biofilms without preliminary binarization. The disclosure of a coverage- and mutant-dependent orientational ordering during biofilm development was advantageously shown at the macroscopic scale.

Moreover, the digital processing approach presented herein and its interpretative scheme not only can be generalized to other organisms but also provides practical guidelines on how to reveal the occurrence of anisotropic self-patterning/ordering in mature biofilms with arbitrary morphological complexity.

## 2. Results

### 2.1. CV-Staining and CFU Counts

**Figure 2:**
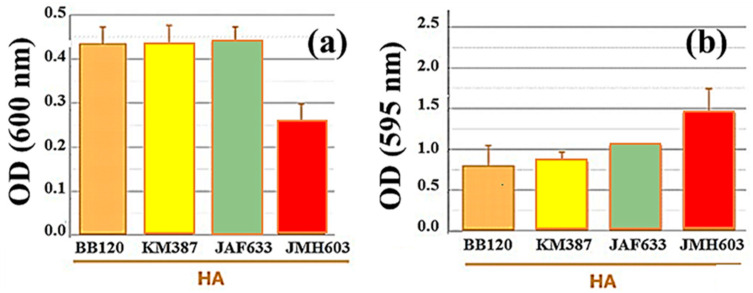
(Color online) Optical density (OD) measurements at (**a**) 600 nm of the planktonic bacteria grown during the biofilm formation experiment and (**b**) at 595 nm to estimate the biomass grown on the HA substrate following CV-staining of the samples.

The number of nonadherent (planktonic) bacteria was calculated by measuring the OD signal at 600 nm of the broth suspension following bacterial growth in the multiwell in the presence of HA substrate. As Figure 2a shows, the highest number of bacteria in the suspension was measured in the case of the wild-type strain BB120 and its mutants KM387 and JAF633, which all showed a similar OD value at 600 nm. On the contrary, the data related to JMH603 indicate a reduction in the planktonic bacteria. The absorbance values at 595 nm (Figure 2b) highlight how the mutant bacteria (KM387, JAF633, and JMH603) grown on the hydroxyapatite produced a biofilm with a higher biomass than the WT (BB120) strain, and the KM387 mutant had a comparable biomass. The strain with the highest biomass was JMH603 followed by JAF633 and, finally, KM387. The results of the CFU/mL (Table 1) confirm these observations, but they do so with some differences. The differences recorded between the various strains are minor, although the increase in the biomass of the biofilms of the mutants was confirmed. The increase in the biofilm production of the JMH603 mutant (*CqsA::Cmr*) and, to a lesser extent, of the JAF633 mutant (Δ*luxM*) was expected since the formation of a biofilm is mainly linked to the late exponential growth phase of the bacteria and is inhibited by the QS AIs that are produced in this phase, such as CAI-I and HAI-1 [24]. Therefore, the lack of production of CAI-I and HAI-1, respectively, in the JMH603 and JAF633 mutants could consequently lead to the stimulation of the production of the biofilms. In addition, the bioluminescence measurements, as an indicator of biomass viability [35] (Table 1), show higher values for BB120 and JMH603 followed by JAF633 and, finally, KM387. The differences recorded among the various *V. campbellii* strains are more evident and puzzling than those in the CFU/mL, although the increase in the biomass of the mutants was confirmed.

### 2.2. Digital Image Processing

The digital processing of the stereomicroscopy images was performed by means of the license-free software FiiJ-ImageJ, which includes plugins for segmentation, quantification, statistics, and data plotting [33,36]. The raw images are matrices of 1280 × 1024 pixels.

**Figure 3:**
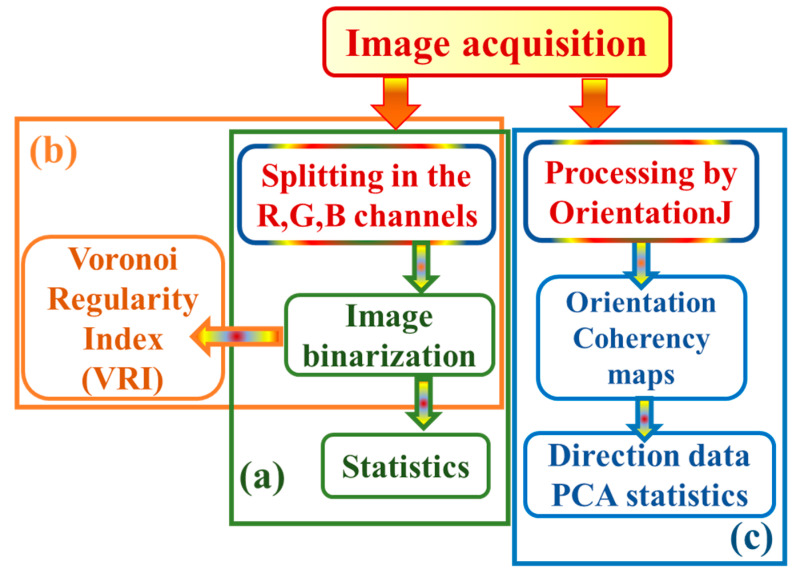
(Color online) Workflow that outlines the applied image processing procedures. Pathway (**a**): the acquired image was binarized and then statistically analyzed to quantify the coverage area of the biofilm. Pathway (**b**): the thresholded image was processed with the Voronoi method. Pathway (**c**): the acquired image was processed with the OrientationJ plugin to obtain orientation and coherency.

Figure 3 sketches the workflow of the image processing procedures implemented in this research, which developed according to three different pathways.

As sketched in Figure 3, pathway (a), the image of interest was split in the associated 8-bit green (G), red (R), and blue (B) color channels to select the best-contrasted grayscale image to be binarized and subsequently processed with the “Analyze particles” plugin of ImageJ, in order to obtain its statistics in terms of count of the objects and their fraction of occupied area. As sketched by the pathway (b) in Figure 3, binary images were also processed through the Voronoi tessellation method to calculate the Voronoi regularity index (*VRI*) [37]. Such an analysis enabled us to quantify the degree of heterogeneity of the biofilm distribution, and, to the best of our knowledge, it has never before been applied to biofilm formations.

Also, the images were analyzed with the semiautomated plugin of ImageJ/Fiji, termed OrientationJ, which is particularly advantageous to investigate very complex morphologies because it does not require preliminary binarization [38]. According to the pathway (c) in Figure 3, the measured images were processed with OrientationJ to obtain orientation and coherency maps over the whole image extension. Then, the direction data resulting from the image processing quantification step were analyzed with the proper statistical tool in the MatLab suite [39].

#### 2.2.1. Thresholding and Statistical Analysis of the Binarized Images

**Figure 4:**
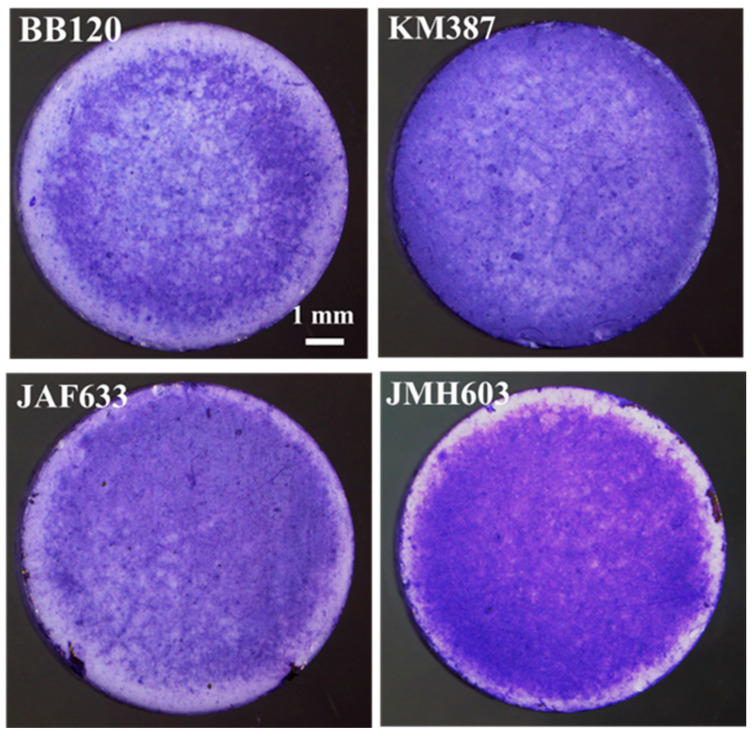
(Color online) From left to right, top to down: stereomicroscopy images of the crystal-violet-stained biofilms associated with the wild-type strain BB120 and its mutants KM387 (Δ*luxS*), JAF633 (Δ*luxM*), and JMH603 (*cqsA::Cmr*).

From left to right and top to down, Figure 4 shows the images of the samples of interest in this study, which were acquired through stereomicroscopy following CV-staining. A visual inspection indicates significant differences in both the degree of coverage, which increased in particular in the case of JMH603, and the morphology of the local features. As a general comment, the biofilm became denser when turning from the wild-type strain BB120 to KM387, JAF633, and JMH603. The lighter features associated with the lower local density of the biofilm exhibited a decreasing size at the increasing of the coverage. In particular, the darker bluish features are associated with the formation of localized colonies.

In order to explicitly and quantitatively assess any differences in the coverage among all the biofilm distributions, the binarization of the images was performed followed by segmentation. The segmentation of bacterial images is usually reported in the case of images with a good contrast between the target features and the background, consisting of small clusters and distributions of sparse bacteria and relatively low coverage. Unlike this standard, our images present a relatively low contrast (that is, a not clearly defined separation between the background and foreground) due to almost high biofilm coverage and complex morphology with overlapping formations that are difficult to be discriminated over the high-coverage regions. All of these aspects are critical and may negatively affect the segmentation process, hence requiring extensive tests and the careful validation of the results. However, the efforts are well motivated because the complex images under consideration are naturally occurring and more informative than the simplified standard ones.

The effective binarization of our complex biofilm morphologies required the extensive implementation of local rather than global thresholding algorithms and the optimization of the size of the computation domain centered at each pixel over the image (see Section 4.4). All the global thresholding algorithms allowed by ImageJ were found to fail due to their yielding binary images with wide dark regions, over-segmentation, or worse replication of details [40,41]. Successful binarization was achieved through systematic local thresholding tests based on all the algorithms allowed by ImageJ with a variable radius (i.e., the size of the local domain over which the threshold is computed). In this regard, we benefited from the observations detailed in [42] about the outcomes of the different techniques. In particular, our extensive tests demonstrated that the local Niblack thresholding algorithm [43] works more accurately and effectively than the others.

**Figure 5:**
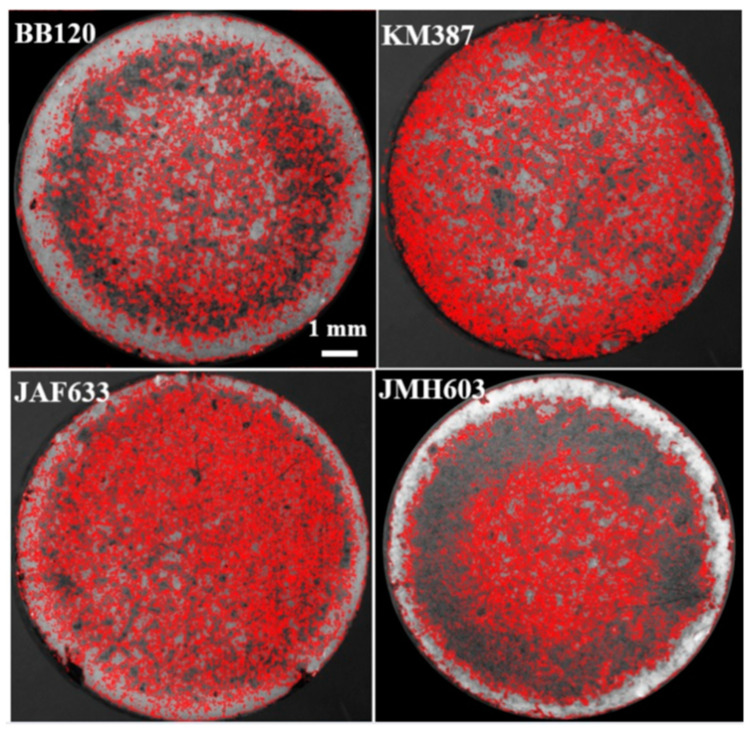
(Color online) Images associated with the four strains BB120, KM387, JAF633, and JMH603 showing the overlap between the segmented objects (resulting from thresholding with the Niblack algorithm and pointed out by red edges) and the input stereomicroscopy grayscale image.

Figure 5 reports the overlap between the red edges of the segmented objects obtained through the Niblack algorithm and the associated stereomicroscopy grayscale input image. Despite the complexity of the images, accurate segmentation was clearly achieved. In the case of the regions associated with lower coverage, the segmented objects fully match the biofilm, and even the fine details, in terms of the edges, were captured through segmentation (see BB120, KM387, and JAF633). The sample showing visually higher coverage, that is JMH603, presents smaller segmented objects in the central region extending to a denser region and going towards the periphery. Accordingly, a decreasing number of counts was expected with respect to the other samples.

All the quantification values associated with the thresholding process with the Niblack algorithm are summarized in Table 2. For each thresholded image, the following values are reported: the radius of the local thresholding domain (R_Niblack_), the number of objects in the segmented image (count), the total area of the segmented objects (A), the percentage of the change in the coverage (relative cvg) of the segmented image with respect to the BB120 segmented image, chosen as the reference sample, and the Voronoi regularity index (VRI).

Effective binarization was achieved with the image-dependent R_Niblack_, and the observed wide variation in the R_Niblack_ has to be ascribed to the morphological differences between the images. The count and area parameters indicate that an increasing number of segmented objects was associated with increasing coverage for BB120, KM387, and JAF633. Differently, JMH603 consisted of a decreasing number of larger objects, hence yielding to a remarkably larger coverage with respect to the wild-type strain BB120 and the other mutans. Indeed, while the relative coverage experienced a slight increase when turning from BB120 to KM387 and JAF633, a remarkable increase (+15.61%) was computed for JMH603. Therefore, the area values in Table 2 highlight a different granularity in the growth and confirm the trend of the quantification obtained through the biological techniques (CV-staining and CFU counting), i.e., increasing coverage in terms of the spatial distribution and density of the mutans compared to the BB120 wild-type strain.

Furthermore, a statistical analysis, in terms of the circularity and aspect ratio of the segmented objects, was performed. Given an object of interest, its shape parameter circularity is defined as

**Figure 6:**
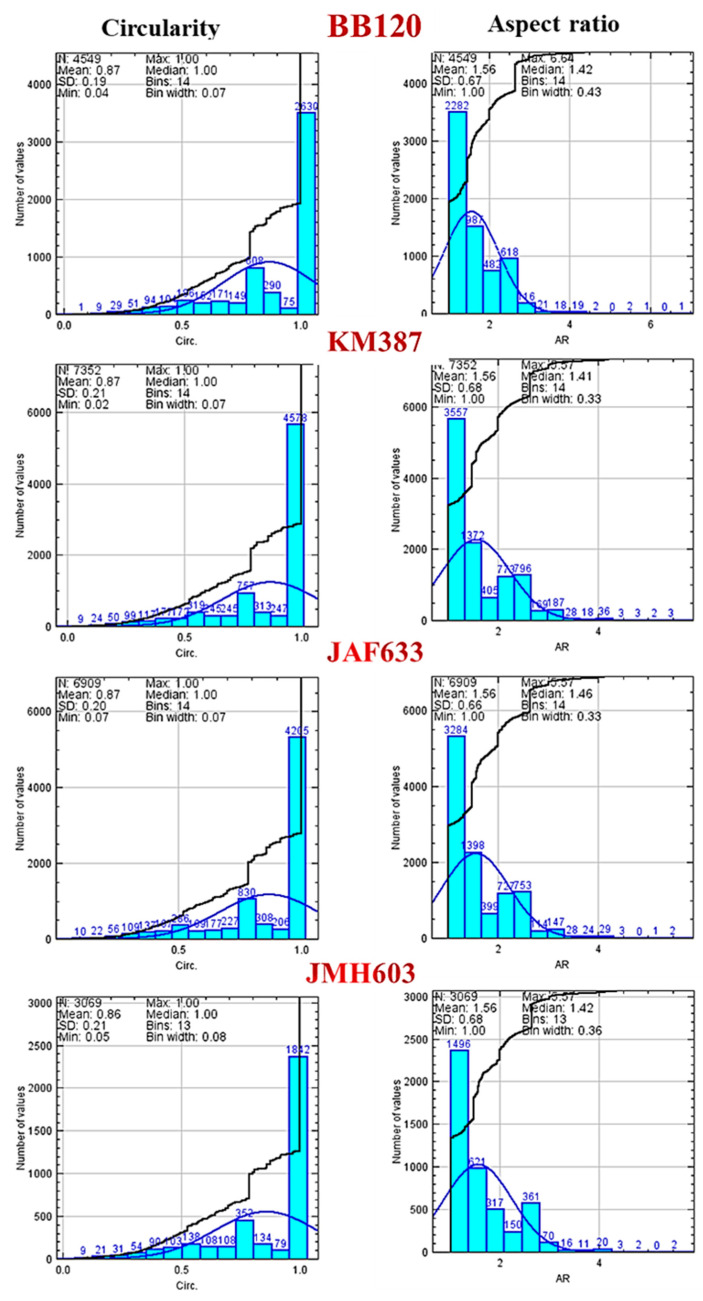
(Color online). From topo to down: histograms of the circularity distributions (Circ, left side) and aspect ratio distributions (AR, right side) associated with the segmented images of BB120, KM387, JAF633, and JMH603.

Circ = 4πA_obj_/P_obj_ (1)

where A_obj_ and P_obj_ are the area and perimeter of the object of interest, respectively, and its aspect ratio can be calculated with the formula AR = major axis/ minor axis. The associated distributions are represented by the histograms displayed in Figure 6, from top to down for each studied image. The optimum number of bins (k) for n data in a histogram was chosen based on the Sturges formula

(k = 1 + 3.322 log n)(2)

to minimize the potential for these pitfalls. According to its definition, the circularity may change due to a change in the area or in the perimeter of the object. An increase in the only perimeter causes the circularity to decrease and corresponds to a decrease in the roundness (surface irregularities) of the object rather than to a deformation [44]. On the other hand, if only the area decreases, the circularity decreases, and the aspect ratio increases because a constant perimeter is associated with the elongation of the object [44]. Therefore, the circularity and aspect ratio provide different information. 

The segmented objects with a Circ ~1 amounted to 58% for BB120, 62% for KM387 and JAF633, and 62% for JMH603 with respect to the total count. The segmented objects with an AR ~1 amounted to 50% for BB120, 48% for KM387 and JAF633, and 49% for JMH603 with respect to the total count. Figure 6 confirms a significantly different behavior for BB120 and JMH603, whilst an almost comparable distribution can be observed for KM387 and JAF633.

As a general remark, the statistical analysis, in terms of the circularity and aspect ratio, would indicate heterogeneous objects, which is consistent with the morphological complexity of the images, and it also suggests that other tools are needed to numerically characterize the homogeneity/heterogeneity of the images more carefully at both the local and global level.

The assessment of the degree of spatial regularity of the segmented objects can be obtained through the Voronoi regularity index (VRI) estimation. In this respect, the data listed in Table 2, which show a very low VRI, despite slight fluctuations associated with the different bacterial strains, would indicate a very low degree of spatial regularity for any biofilm distribution under consideration. Of note, a comparable VRI between BB120, which had the lowest coverage, and JMH603, which had the highest coverage, suggests that the average of the areas of the Voronoi cells used to calculate the VRI may impact the final result in terms of sensitivity to the local characteristics of the image that were hidden due to the global information.

Therefore, to shed light on the small perturbations in the frequency of the shortest nearest neighbor distances, a deeper analysis at the local level has to be performed. Although the Voronoi method can be applied locally to an ROI, it suffers from the drawback that it requires the preliminary binarization of the image. As discussed, besides being very difficult, binarization may be prone to significant uncertainties, especially for particularly complex morphologies and low-contrast images due to high coverage, which makes it difficult to discriminate the background and foreground. To overcome such limitations, the numerical description of the homogeneity/heterogeneity of our images should exploit the original source image. Based on this reasoning, local computation using the structure tensor implemented in OrientationJ was considered.

#### 2.2.2. Analysis of the Images: Directional Correlation over Subdomains

The structure tensor detects the predominant directions or the anisotropy in a given neighborhood of a pixel in an image and the degree of coherency of those directions. The OrientationJ plugin provides a number of gradient operators (cubic spline, finite difference gradient, finite difference Hessian, Fourier gradient, Gaussian gradient, and Riesz filter) that were all tested on our images to assess their associated degree of accuracy. In our case, the best performing gradient operator, in terms of trade off for speed and accuracy, was found to be the Riesz filter. It is a translation-, rotation-, and scale-invariant transformation with the advantage of reducing image noise and avoiding the amplification of high frequencies. The successful output allowed through the application of the Riesz filter to our images can be ascribed to the occurrence of low-contrast structural differences that make the other gradient operators unsuitable for an accurate estimation of the image coherency.

**Figure 7:**
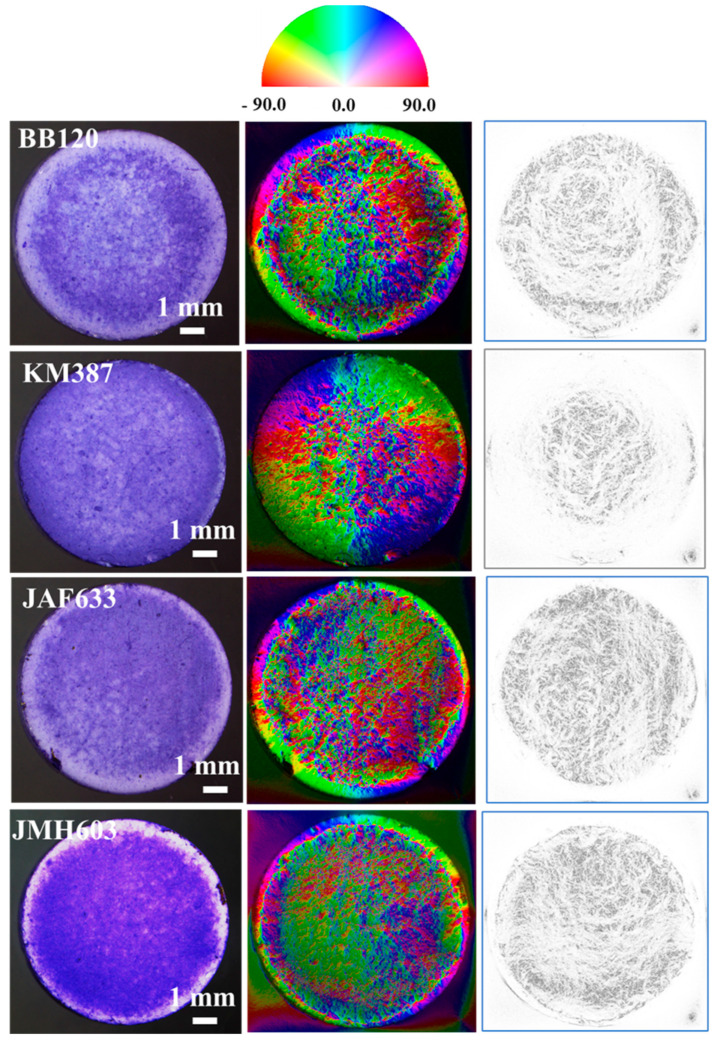
(Color online) From top to down: results of the OrientationJ-based analysis using the Riesz filter on all the considered images. Left column: stereomicroscopy images of interest. The middle and right columns show the hue–saturation–brightness (HSB) color-coded maps and the coherency maps, respectively. The color wheel is also reported on the top.

**Figure 8:**
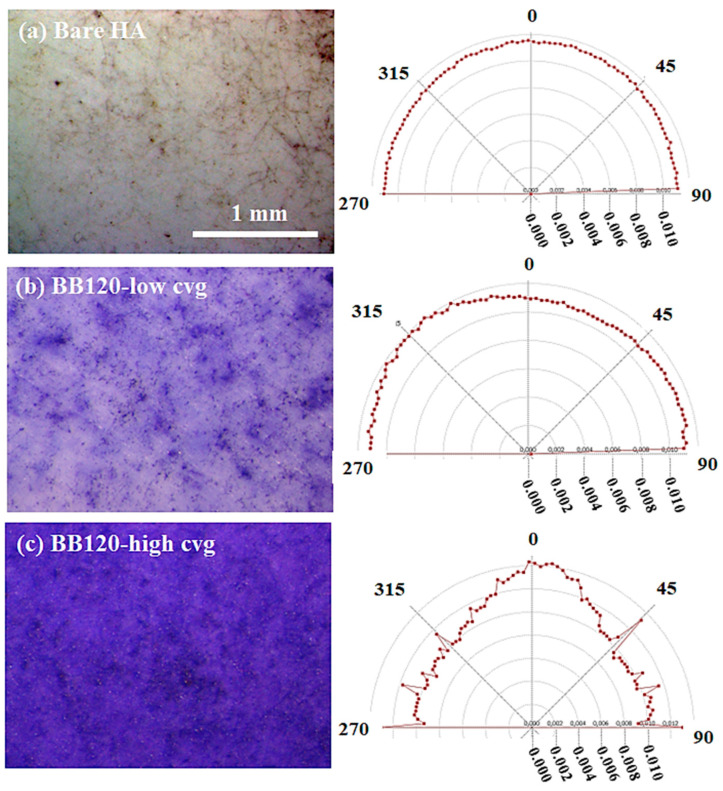
(Color online) From top to down, left column: analysis of the occurrence of orientations in the case of comparable areas belonging to the bare HA (**a**), low-coverage BB120 (**b**), and high-coverage BB120 (**c**). The associated polar plots of the orientations are reported on the right column.

Figure 7 reports the stereomicroscopy images of interest in our discussion on the left-side column and the associated hue–saturation–brightness (HSB) color-coded maps (middle column) and grayscale coherency maps (right-side column) resulting from the application of the Riesz filter to our images. The Riesz filter maps are color-coded according to the orientation, as described in the color wheel, of the domains they belong to. They show the occurrence of the local orientational correlation in the subdomains distributed over the whole surface of the sample and characterized by a few dominant orientations. As it will be extensively discussed, relevant differences were observed among all the investigated strains (wild-type strain and mutants). For the coherency maps, the brighter regions (high coherency) indicate local orientation, and the darker regions (low coherency) indicate isotropy. Of note, since zero coherency was never observed, meaning that the darker regions are associated with gray levels, some degree of local orientation occurred over the whole image. In order to gain more insight in the isotropy/anisotropy and coherency properties of our images, the OrientationJ-based analysis was performed over either a bare HA substrate, to exclude any influence on the observed orientations of the biofilm growth, or ROIs which were chosen according to the main criterion of increasing coverage. Figure 8 compares the results of the orientational investigation performed over ROIs with comparable size belonging to the bare HA substrate (Figure 8a) and both low-coverage (Figure 8b) and high coverage (Figure 8c) biofilm formations of the wild type strain BB120. The polar plots exhibited no preferred direction in the case of the bare HA.

For the BB120 samples in the topmost line of Figure 7, directionality properties can be clearly observed and were especially enhanced for increasing coverage. These findings let us conclude that HA substrate is not responsible for the oriented microdomains disclosed for the biofilms under study and that coverage plays a role in driving the local orientation of the growth of *V. campbellii* and the mutants studied in this paper.

**Figure 9:**
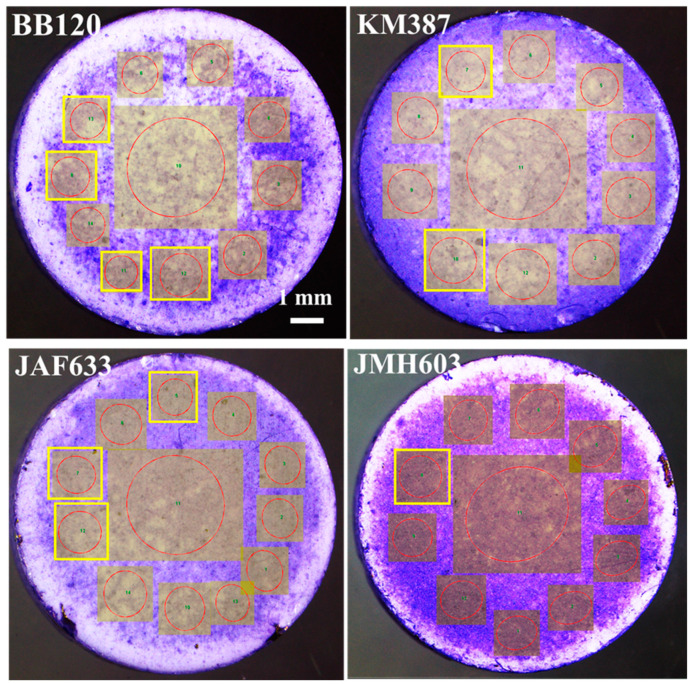
(Color online) From top to down, left to right: results of the OrientationJ-based analysis of the *V. campbellii* wild-type strain BB120 and its isogenic derivatives JAF633 (Δ*luxM*), KM387 (Δ*luxS*), and JMH603 (*cqsA::Cmr*) performed over a number of ROIs (darkish squares). The occurrence of a local preferred orientation is pointed out by elongated red circles. Yellow rectangles point out the ROIs associated with circles, referring to isotropy.

Since even a direct visual inspection of the stereomicroscopy images in Figure 4 clearly points out that the biofilm coverage increases when moving from the center to the periphery of the sample, differences in the results of the Orientation-J analysis between these different locations in the samples were investigated. The results of the local analysis of directions performed for each sample and shown in Figure 9 demonstrate the occurrence of directionality through the slightly deformed red circles associated with the ROIs highlighted by the darkish squares drawn over the stereomicroscopy image. The yellow squares highlight the local ROIs exhibiting no or very weak directionality. The contrast/brightness of the measured images were modified in such a way to highlight the local ROIs and the associated red circle-like contour. To clarify, red contours were drawn from OrientationJ for each local selection (ROI) and measure the occurrence of a directionality through their degree of elongation (ratio of major to minor axes) and elongation direction. Hence, elongated ellipses refer to a local orientation as well as high coherency. Circles refer to the isotropy or poor anisotropy properties of the subdomain under analysis. It can be clearly observed that red circle-like contours were mainly associated with the central ROI, except for JMH603, which shows a red ellipse for the extended ROI around the center of the sample. Of note, for BB120, JAF633, and KM387, the central ROI was associated with a not perfect circle, meaning that a weak directionality occurred. Since JMH603 had higher coverage than the other samples, this finding supports the fact that increasing coverage drives the formation of oriented patterns. On the other hand, low aspect ratio ellipses can be observed along the periphery of the samples with number density and elongation, which increased when turning from BB120 to JMH603, referring to increasing coverage again.

#### 2.2.3. Statistical Analysis

**Figure 10:**
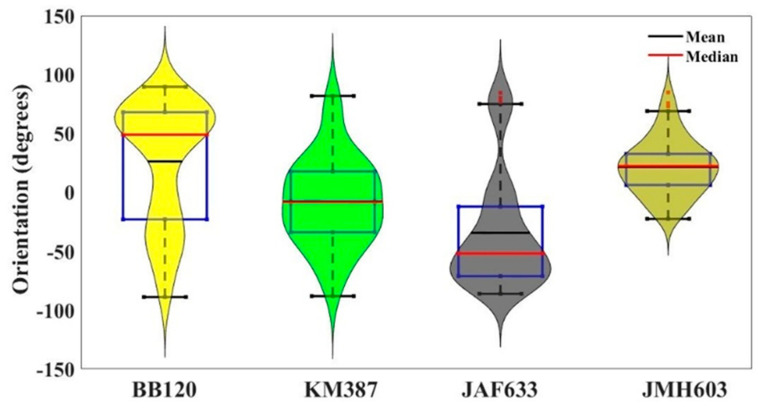
(Color online) Boxplot of the distribution of the orientation angles for each considered image superimposed on a violin plot. The central red mark indicates the median, and the blue bottom and top edges of the box indicate the 25th and 75th percentiles, respectively. The whiskers extend to the most extreme data points not considered to be outliers, and the outliers were plotted individually using the red “square” symbol. The violin plot depicts the distributions of the observed directions in terms of density curves (*see the text*).

In this section we present and discuss a series of statistical analyses. First of all, we drew 20 ROIs (250 × 250 pixels^2^ corresponding to a 2.0 × 2.0 mm^2^ area) on each of the stereomicroscopy images of (1800 × 1800 pixels^2^), in such a way the entire surface of the image was covered. For a given sample and for each ROI, the angle values, as provided by the OrientationJ measure tool, were recorded. We collected a series of N = 60 orientations for each sample and calculated the associated statistics. To give a complete description of the orientations of the BB120 strain and its mutants, Figure 10 presents a violin plot with a superposed boxplot representation of all the directions exhibited by the *Vibrio* bacterial strains under analysis [40,41]. A violin plot depicts distributions in terms of density curves to better highlight the “shape” of the distribution, including the peaks of frequently occurring values and the skewness too. The width of each curve corresponds with the approximate frequency of the data points in each region. Outliers, i.e., values amounting to more than 1.5 times the interquartile range or approximately 3 standard deviations in a Gaussian distribution, were dropped out. The boxplot in Figure 10 shows the central mark (red line) on each box, which indicates the median, and the bottom and top edges of the box indicate the 25th and 75th percentiles, respectively. The whiskers extend to the most extreme data points not considered to be outliers, and the outliers are plotted individually using the red “square” symbol. It is clearly evident that all the distributions significantly differed from each other; KM387 and JMH603 had a more compact shape (meaning a minor spreading of orientations) even if many outliers were present for JMH603. The BB120 strain and the JAF633 mutant had a larger distribution with bimodal behavior, and a major number of outliers.

In conclusion, the presented analysis disclosing local orientations is not only consistent with the above discussion linked to the defined orientational phenomena with increasing coverage but also, and more remarkably, confirms a biological statement, i.e., the BB120 strain and its mutants are really specialized with different peculiarities.

At this point, a number of statistical evaluations were performed in order to numerically assess the correlation/connection between the orientation angles of the wild-type strain and its mutants as well as between all the mutants. In this respect, Pearson and Spearman correlation statistics were implemented, where Pearson correlation indicates a linear relationship between two series of data and where Spearman correlation is associated with a monotonic relationship. The calculation of both the Pearson and Spearman correlation coefficients allowed us to observe that all the mutants weakly correlated with each other and with the BB120 strain. As a remark, for BB120 vs. JAF633 and JAF603 vs. KM387, the correlation was negative (−0.0073 and −0.096 Pearson values, respectively), which refers to a certain degree of sensibility of the index to different phenomena, and this was opposite to all the other correlations, which exhibited positive correlations. However, all these correlations were not statistically significant (*p*-value > 0.05).

Thus, we calculated the ꭓ^2^ value to estimate the goodness of the fit after rescaling all the angle values in the range of [0,180] degrees in order to avoid negative values. We observed a weak connection between all the strains but a statistically significant difference (*p*-value << 10^−^^6^) between JMH603 vs. BB120 and JMH603 vs. KM387.

Finally, to obtain decisive confirmation of a mutation-dependent orientational patterning of growing biofilm, we performed a principal component analysis (PCA), which is a powerful and common technique in fields such as face recognition and image compression or population genetics, microbiome studies, and atmospheric science. It advantageously works to find patterns in high-dimensional data by linearly transforming the initial data into a new coordinate system where (most of) the variation in the data can be described with fewer dimensions than the initial system, allowing the visual identification of the clusters of closely related data points [42].

**Figure 11:**
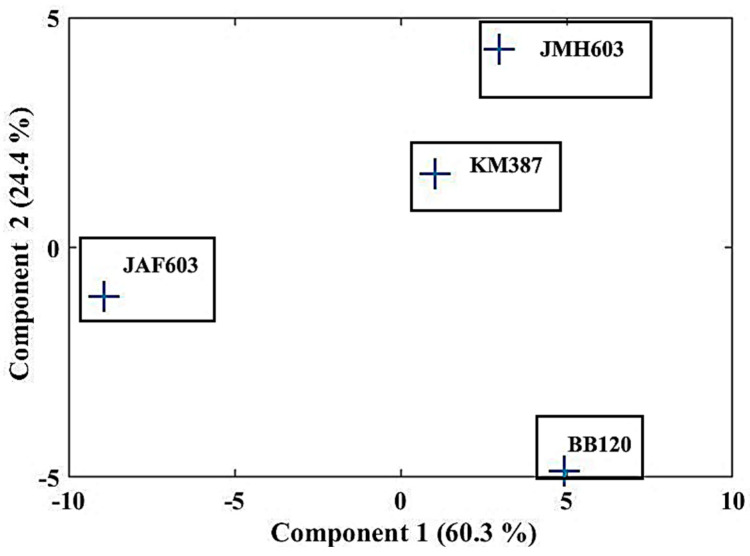
(Color online) Principal component analysis (PCA) of the orientation angles of the *V. campbellii* wild-type strain BB120 and its isogenic derivatives JAF633 (Δ*luxM*), KM387 (Δ*luxS*), and JMH603 (*cqsA::Cmr*). Configurations resulted to be well distinguishable and very far from each other.

Indeed, the main advantage of this technique is that, once certain patterns are found in the data, especially when samples are high-dimensional, it is possible to compress the input space, i.e., to reduce its number of dimensions, with a modest loss of information in describing the whole system. From the boxplot (Figure 10), it was evident that some variables (BB120 and JAF603 in particular) had greater variance than the others. Since PCAs are based on maximizing variance, i.e., are quite sensitive to the variance in the initial variables, they may have a certain impact on biasing the first principal component. Therefore, we preferred to make the PCA as independent as possible of such a situation by standardizing all the variables so that each of them contributed equally to the analysis. In other words, by means of normalization, we transformed the initial values to be closer to a Gaussian distribution (mean = 0 and standard deviation = 1). Thus, after constructing a vector of features comprising all the orientation angles, we obtained that the first and second components explained about 85% of the information; thus, the orientations could be well differentiated (Figure 11). Figure 11 makes it evident that the angles follow distinctive patterns with well-separated positions in the space of the components and greatly different distances among them. Specifically, the closest behavior was highlighted between BB120 and the JMH603 mutant but was very far from JAF633 and KM387. This enforces the conclusion addressed by the evaluation of ꭓ^2^ for stimating the goodness of the fit, confirming a mutation-dependent patterning of the orientation.

## 3. Discussion

To get started, the thresholding and estimation of the fractional coverage area were obtained through the extensive application of global and local thresholding algorithms. In this step, the morphological complexity of the images and the impact of high coverage on their contrast was discussed to play a relevant role in making binarization challenging. As a result of extensive tests, the quantification of the biofilm coverage, consistent with and supporting the biological counts, was obtained.

In order to gain further information on the local morphology of the biofilm formations, Voronoi tessellation, commonly used to study crystals and defects in crystals, was applied as a general method able to track ordering at either a long or short range. The main conclusions were that the method may suffer from segmentation-related inaccuracy, which may be critical when complex morphologies have to be digitally processed. Moreover, it was discussed that the estimation of the Voronoi index may be not decisive in assessing the presence of ordering in the case of high-coverage mature biofilms.

To overcome the limits and difficulties in thresholding unconventionally complex images, we processed the raw images rather than the binarized ones, demonstrating the remarkable benefit of our approach. Since our analysis was performed on measured images, processing related artifacts, modelling, and/or complex binarization were avoided. Hence, very importantly, the features associated with the real images were retained and pointed out. Therefore, the developed image processing procedure was revealed to be decisive in addressing the main outcome of this study, that is, the occurrence of oriented subdomains in each sample under consideration. An interesting aspect that was worth being studied was if and how the observed orientational patterning was mutant-dependent. Statistical correlation and PCA techniques were demonstrated to be suitable for addressing this topic.

In the next, several points of peculiar characteristics of the observed results and developed analysis will be remarked and compared with the literature.

### 3.1. Modelling Biofilm Early Development

In the literature, individual-based models (Ib-Ms) are general approaches for modelling biofilm development that describe the characteristics of individual bacterial cells represented as spherical particles [43]. More recent models account for the most realistic rod-like shape of bacterial cells, which introduces the dependence of biofilm development on the aspect ratio, polar growth, and the influence of the division patterns on the cell geometry and critical length [45,46]. These models enable one to describe the internal organization of growing colonies, as they are related to the cell-to-cell interactions, and provide information on the development of front roughness and cell alignment in the biofilm [45,46].

### 3.2. Orientational Phenomena in Growing Biofilms

In the literature, it is documented, for instance, the occurrence of clusters with orientational organization while modelling the early biofilm microcolony formation of the Gram-negative bacterium Pseudomonas putida with a Brownian-dynamics-based IbM mathematical model [47]. Clusters having different degrees of internal and orientational order were shown to form as a function of the aspect ratio of the individual rod-shaped bacterial cells and the relationship between the growth and diffusion rates. Simulations indicate that the predominance of growth over diffusion combined with large aspect ratios favored the internal organization, consisting of bacterial cells arranged in similarly oriented domains, where orientation refers to vertical cells (i.e., cells aligned side by side and oriented vertically to the supporting surface) and horizontal cells (i.e., cells aligned side by side and oriented parallel to the surface). Several constraints were introduced in the modelling process: (i) a study of the early stages of growth was performed, (ii) the biofilm formed on a surface exposed to a liquid phase, (iii) an exchange of cells between the biofilm and the bulk liquid phase as well as active motility on the surface were ruled out, (iv) there was a constant growth rate and diffusion rate, and (v) there was no impact of the genetic factors and mutations on the colony organization.

As a further example, modelling studies of *Vibrio cholerae* report differential growth consisting of cell verticalization (i.e., cells aligned side by side and oriented vertically to the surface) in the core that drives and is affected by the radial alignment in the periphery in the presence of adherent mutants [14,15]. Additionally, for the parent strain of the *V. cholerae* biofilm, a higher local volume fraction was demonstrated to be correlated with a stronger local alignment [15]. Other studies report the cell-to-colony dynamics and structural evolution of a growing biofilm to be affected by the cell–surface interaction and confinement conditions. These aspects highlight the role played by bacteria in shaping the biofilm morphogenesis and to bridge the interactions at the cellular scale with the biofilm patterns at the community scale [11,13,14,15,16]. These investigations exploit high-resolution imaging and computationally demanding simulations, due to the need to identify single bacteria in small and growing clusters. In these cases, thresholding and segmentation processing are not prohibitive and enable one to discriminate as precisely as possible vertically from planarly oriented bacteria.

With respect to the literature, our study does not aim to track the features linked to individual cell growth or orientation, in vertical or horizontal alignments. On the contrary, it investigates on the occurrence of orientational behavior at the macroscopic scale. The outcome indicates a particular contribution for each mutant, suggesting that the mechanism yielding the orientational features could be not only due to the interplay between the growth and diffusion rates (i.e., purely physical factors). This aspect deserves further investigation.

### 3.3. Isotropy/Anisotropy Properties at the Macroscopic Scale

Of note, the occurrence of the mutant-dependent short-range directional and coherent development of the biofilm pattern would be unthinkable when considering only a visual inspection of the stereomicroscopy images. Despite the fact that the visual inspection of the images would indicate the anisotropy of the mature biofilm formations, subdomains of the images with preferred directions were detected. Interestingly, the deep digital processing and statistical analysis of the complex mature biofilm images allowed us to highlight the presence of an orientational correlation for all the investigated bacterial strains. In practice, the performed image processing and statistical analysis of the orientation distributions for each sample indicated a coverage- and mutant-dependent orientational ordering during biofilm development that was advantageously shown at the macroscopic scale.

### 3.4. Light Microscopy Tool

From the operative point of view, since our study develops the investigation of the occurrence of orientational behavior at the macroscopic scale, biofilm imaging through light microscopy can provide useful and direct information about the impact of specific conditions (in this case, the specific mutant) on biofilm growth or on triggering self-patterning and morphological changes.

### 3.5. Digital Image Processing: Methodological Contribution

On the other hand, whereas the applied workflow combines already existing software tools and statistical methods, a novel methodological contribution is provided that explains how to apply the existing knowledge to study a biophysics/biomedicine problem that is characteristic of complex mature biofilms over large areas by overcoming the prominent limitations of common techniques that rely on the segmentation and modelling of simplified and unrealistic bacterial communities. The developed approach has general applicability, extends the field of application of biomedical informatics, and enables progress in obtaining knowledge on a wide range of cases that are complex or prohibitive to being studied computationally.

### 3.6. Contrast Enhancement

For the sake of honesty, we have to report that, whenever raw light microscopy images are not optimally contrasted (due to an inhomogeneous background, noise or halos, measurement-related artefacts, or imperfect focusing related to the tridimensionality of the sample), the discrimination of features can be particularly difficult. Moreover, mature biofilms being tridimensional high-density formations, the gradient approach may suffer from a reduced degree of accuracy in detecting the local orientational behavior. Under these situations, the preliminary enhancement of the image, which may not be straightforward [48], is demanding, even if thresholding and binarization is not required.

### 3.7. Pros and Cons of Automated Methods

Finally, even though our analysis procedure was not automated, meaning that the user has to manually process each image by implementing a number of analysis tools offered by OrientationJ and exporting the data file for subsequent statistical calculations, this aspect does not constitute a crucial limitation. Although a nonautomatic procedure may be annoying and time-consuming, it represents a benefit for a skillful and curious user who can make a proper decision about what to investigate and the size of the ROI depending on the characteristics of the image and research purposes. Anyway, the information that can be gained compensates the user’s efforts, because biofilm development on patterned and unconventional substrates can be studied over extended surfaces with a localized analysis (meaning by setting size and location of a ROI). Moreover, the proposed analysis does not demand the acquisition of large dataset of high-resolution images.

## 4. Materials and Methods

### 4.1. Vibrio campbelli Strains (Wild-Type Strain and Mutants)

The QS circuit of *Vibrio campbellii* is sketched in Figure 1 and was reworked from Anetzberger et al. [31]. In this bacterial species, three AIs, referred to as HAI-1, AI-2, and CAI-1, are synthesized by three synthetases (*LuxM*, *LuxS*, *and CqsA*) [22,24].

Four different *Vibrio campbellii* (*Harveyi* clade) strains were used in this study and were kindly provided by Prof. Bonnie L. Bassler (Princeton University, Princeton, NJ, USA). Specifically, the *Vibrio campbellii* strain BB120 (wild type, also known as ATCC BAA-1116) and its isogenic derivatives JAF633 (Δ*luxM*), which lacks in AI-2; KM387 (Δ*luxS*), which lacks in HAI-1; and JMH603 (*CqsA::Cmr*), which lacks in CAI-1 [22,24], were used. The related hybrid sensor kinases (*LuxN, LuxP/LuxQ, and CqsS*) measure the levels of the AIs. With the high AI concentration, the autophosphorylation activity of the hybrid kinase is inhibited. Phosphate groups are transferred to the sigma54-dependent activator of transcription, *LuxO*, through a phosphate layer including the histidine phosphotransfer protein *LuxU*. *LuxO* activates the transcription of Qrr1-4 regulatory sRNAs, which, together with the RNA chaperone *Hfq*, controls the transcript encoding the master regulator *LuxR*. Genes required for bioluminescence, biofilm formation, and proteolysis are activated by the AIs. On the contrary, they repress other genes (type III secretion and siderophore production).

### 4.2. Biofilm Growth

The bacteria were grown in Petri dishes containing luminescence agar medium (LA), which were incubated at 28 °C for 18 h. Subsequently, a colony was removed and inoculated in 10 mL of liquid LA. The inoculum was incubated at 28 °C with shaking (180 rpm). Biomass was measured using optical density (OD) at 600 nm (ONDA spectrophotometer). When the OD datum was 0.3–0.5, an aliquot of 0.5 mL was withdrawn and used to inoculate 50 mL of liquid LA (dilution: 1:25). This bacterial suspension was used to set up the bioluminescence experiment and for growth experiments of biofilm formations. In this respect, hydroxyapatite (general formula: Ca_10_[PO_4_]_6_[OH]_2_) (HA) discs (diameter (D) of ~1 cm, bonding chemical) were preliminarily autoclaved at 121 °C for 20 min and were placed in the wells using sterile forceps. The bacterial suspension was used to fill each well of a 24-well sterile multiwell (CytoOne^®^) with 1 mL. The multiwell was incubated at 28 °C under agitation (120 rpm) until the end of the experiment. Bacteria were allowed to grow on HA culture substrate for 48 h at 37 °C. Of note, we did not consider the temporal evolution of the biofilm growth because we were interested in studying the morphological and isotropy/anisotropy properties of biofilms in a mature state. This issue was particularly interesting because growing bacterial biofilms may present a number of surface morphologies strongly affected by physicochemical conditions [11,12]. Moreover, investigation of realistic and complex mature biofilm morphologies requires different methods with respect to conventional distributions, including sparse bacteria and small clusters/colonies. The bacterial formations grown on HA substrates were used to carry out various measurements to estimate the biomass adhering to the support. On the other hand, OD measurements at 600 nm were conducted on the suspension liquid medium above the HA substrate to estimate the biomass of the planktonic cells.

Hydroxyapatite multicomponent systems are widely used in the medical field due to inertness, long-term life, biocompatibility, and possibility to modify bacterial adherence and enhance antibacterial surface activity through nanostructuring as well as through doping/coating with metal ions (Mg^2+^, Sr^2+^, Zn^2+^, Cu^2+^, Ag^+^) or functionalization with antibiotics [49,50]. We considered pristine HA to be an ideal inert substrate with no impact on the growth of the biofilm; hence, the role of the mutants could be highlight at best.

Bioluminescence was measured using a luminometer (Turner BioSystems) at the end of experiment (48 h of growth).

### 4.3. Biomass Measurements: Crystal Violet Staining and Colony Count

Biomass adhering to the supports was quantified by using a protocol based on crystal violet (CV) staining [51] and was modified as follows. Following growth, the LA broth was removed, and the supports were washed 3 times in a sterile saline solution (0.9% NaCl) and were immersed in methanol (100%) for 15 min to fix the bacteria. Then, the HA supports were stained through immersion in a solution of CV (diluted with 8 volumes of water) for 5 min [52]. Multiple replicated, stained, and dried samples were prepared for imaging with stereomicroscope (Nikon, SMZ 1270, zoom ratio of 12.7:1, zooming range of 0.63–8×) and for colony count experiments. For this purpose, CV-stained supports were washed 3 times in the sterile saline solution and were then treated for 15 min with ethanol (96%) to detach the bacteria biomass. Optical density at 595 nm of the ethanol-based whitening solution was measured. Multiple (replicated) media were used for each test. Each experiment was also repeated using microorganism-free media (blank). This protocol measured all biomass attached to the material, including both dead and living bacteria and the extracellular matrix.

Following growth, colonies were digitally counted and analyzed. Data were expressed as percentage values of colony forming unit (CFU) linked to bacterial cells grown on regular agar plates. The colony count protocol (CFU/mL) was used to measure live bacteria only. The supports were washed 3 times in sterile saline and were placed in 15 mL tubes filled with 2 mL of LA. Each tube was vortexed for 2 min. From each sample thus obtained, a 100 µL aliquot of medium was taken from the test tube and was diluted 4 times from 1 to 10, and, thus, 10 µL were taken and subsequently seeded on an LA agar plate for each dilution.

### 4.4. Digital Image Processing

#### 4.4.1. Preprocessing of the Images: Binarization and Statistics

Binarization is the image analysis process that permits of discriminating between two classes consisting of different objects, as associated with background or foreground. The most common technique is based on setting a threshold value to classify the pixels that form the image in two regions/classes, including the pixel values falling below or above the threshold. Furthermore, single or multiple thresholds can be defined in order to obtain greater accuracy in discrimination [53]. The final outcome is a binarized image in which 0/1 px value refers to belonging or not belonging to a particular class of interest. Because of the morphological complexity of our images, thresholding was particularly critical. Global thresholding algorithms allowed by ImageJ (that is, Default, Huang, Huang2, Intermodes, Isodata, Li, Max Entropy, Mean, MinErr, Minimum, Moments, Otsu, Percentile, Reny Entropy, Shanbhag, Triangle, and Yen) were found to fail due to their yielding binary images with wide dark regions, oversegmentation, or worse replication of details [54]. Successful binarization was achieved through systematic local thresholding tests based on all the algorithms allowed by ImageJ (i.e., Bernsen, Contrast, Mean, Median, MidGray, Niblack, Otsu, Phansalkar, and Sauvola) with a variable radius (i.e., the size of the local domain over which the threshold is computed). In this regard, we benefited from the observations detailed in [48] about the outcomes of the different techniques. In particular, our extensive tests demonstrated that the local Niblack thresholding algorithm [55] worked more accurately and effectively than the others. It set the threshold value to each pixel-wise by sliding a rectangular window with tunable size over the gray-level image [55]. The threshold value (*thr_Niblack_*) is given by the formula

*thr_Niblack_* = *m*(*x*,*y*) + *k* × *S*(*x*,*y*) (3)

where *m* and *S* stand for the mean and standard deviation of all the pixels in the local rectangular domain around (*x,y*), respectively, and where *k* is an empirically fixed parameter that may be −0.1 or −0.2 depending on the background noise. In our local thresholding tests, the radius of the local domain yielding the best segmentation issue was found to be image-dependent. This was consistent with both the morphological complexity of the images under consideration and the fact that the size of the neighborhood surrounding the pixels in (*x,y*) varied, constrained to be either small enough to retain local details or large enough to minimize noise.

#### 4.4.2. Voronoi Method

In image analyses, one of the most important tools for understanding the nature of the patterning in a two-dimensional array of objects is a Voronoi tessellation, which provides an immediate visual sense of the degree of regularity within the mosaic, which is conveyed by the variation in the size of the Voronoi cell areas [56]. Voronoi tessellation is a geometrical method that partitions the space through the assignment of seed points and grouping of points in the space between and around seeds according to the criterion of the closest seed. Briefly, given a set, *P-{p_1_,….,p_n_}*, of generators, a Voronoi tessellation is defined as a subdivision of the space into *n cells*, one for each site in *P*, with the property that a point *q* lies in the cell corresponding to a site *pi* if *d(pi, q) < d(pj, q)* for *i* ≠ *j* in some metric (often the Euclidean metric). Hence, given a set of *n* seeds, the Voronoi tessellation technique generates a partition of the space in *n* polygonal regions, differing in shape and size depending on distances among all neighboring seeds and isotropy/regularity of the distribution of the seeds. This partition and polygons are termed Voronoi diagram and Voronoi cell, respectively. Each Voronoi cell associated with a given point includes points that are closer to it than any other point. Let *D* be average distance between the centroid of each Voronoi cell and its vertices; then, the *VRI* is defined as the ratio of *D* divided by the standard deviation associated with *D*. The meaning of the *VRI* is the variation in nearest neighbor distance with respect to its average value, and it is a measure of the regularity of the distribution. For instance, large *VRI* is associated with regularity, and *VRI* approaching zero is indicative of poor regularity/randomness [57].

#### 4.4.3. Image Processing Based on OrientationJ

OrientationJ was developed to characterize isotropy properties of a region of interest (ROI) around a given point (*xo,yo*) based on the local structure tensor, that is, a matrix derived from the gradient of the image [34]. The 2D structure tensor associated with a function of 2 variables f(x_1_,x_2_) is defined as follows:

(4) $${J(x_{0})}_{w}=\iint^{\;}w\left({x-x}_{0}\right)\left(\begin{array}{cc} f_{x_{1}}^{2}\left(x\right) & f_{x_{1}}\left(x\right)f_{x_{2}}\left(x\right)\\ f_{x_{2}}\left(x\right)f_{x_{1}}\left(x\right) & f_{x_{2}}^{2}\left(x\right) \end{array}\right){\mathrm{dx}}_{1}{\mathrm{dx}}_{2}$$

where w(x, y) ≥ 0 is a weighting function that is associated with the ROI (a smoothing filter, usually Gaussian) and where f_xi_ is the partial derivative of a function f calculated with respect to the variable xi (i = 1, 2). It encodes the local orientation and anisotropy of an image based on the orientation specified by the angle θ (associated with the largest eigenvalue of the structure tensor)

(5) $$\theta =\frac{1}{2}\mathrm{arctg}\;\left(2\frac{\langle f_{x_{1}}{|f}_{x_{2}}\rangle {}_{w}}{\langle f_{x_{2}}{|f}_{x_{2}}\rangle {}_{w}-\langle f_{x_{1}}{|f}_{x_{1}}\rangle {}_{w}}\right)$$

and the coherency index C, ranging from zero to unit, which provides a degree of directionality at any point, is given by

(6) $$C=\frac{\;\sqrt{{\left(\langle f_{x_{2}}{|f}_{x_{2}}\rangle {}_{w}-\langle f_{x_{1}}{|f}_{x_{1}}\rangle {}_{w}\right)}^{2}+4\langle f_{x_{1}}{|f}_{x_{2}}\rangle {}_{w}}}{\langle f_{x_{1}}{|f}_{x_{1}}\rangle {}_{w}+\langle f_{x_{2}}{|f}_{x_{2}}\rangle {}_{w}}$$

where

(7) $${\langle f|g\rangle }_{w}=\iint^{\;}w\left(x_{1}{,x}_{2}\right)f\left(x_{1}{,x}_{2}\right)g\left(x_{1}{,x}_{2}\right){\mathrm{dx}}_{1}{\mathrm{dx}}_{2}$$

is the weighted inner product [58]. While the structure tensor lets one detect low-order characteristics, higher-order directional structures require higher-order derivatives, such as, for instance, the square matrix of second-order partial derivatives of a function (Hessian filter) or *n* > 2 derivatives.

In the case of OrientationJ-based processing of an image, f(x_1_, x_2_) is the histogram intensity of the gray levels associated with each pixel of the image, and the structure tensor is calculated along the principal spatial directions x and y. The user specifies the size of a Gaussian-shaped window to be translated over the entire image for each pixel of the image and the gradient operator for the calculation of orientation (cubic spline, finite difference gradient, finite difference Hessian, Fourier gradient, Gaussian gradient, and Riesz filter are allowed). As a result of computing local orientation or anisotropy and coherency index, color images with orientation typically encoded in the HUE color appearance (hue–saturation–brightness map, where hue is orientation, saturation is coherency, and brightness is the input image) and gray level coherency maps are obtained [59].

## 5. Conclusions and Future Perspectives

In this study, we reported a general and easy-to-implement digital image processing approach able to disclose the orientational correlation of the mature high-coverage biofilms of the *V. campbellii* wild-type strain BB120 and its isogenic derivatives JAF633 (Δ*luxM*), KM387 (Δ*luxS*), and JMH603 (*CqsA::Cmr*). Images were provided through low-cost and commonly available light microscopy (stereomicroscopy) following bacterial growth on hydroxyapatite substrates.

The morphological complexity and the coverage-related low contrast of the images under consideration were critical to the thresholding and estimation of the fractional coverage area to be compared with quantification obtained through biological procedures. Accomplishing these goals required the extensive and time-consuming application of global and local thresholding algorithms. Then, Voronoi tessellation was demonstrated to be critically affected by segmentation-related inaccuracy and to not be decisive in assessing the presence of ordering due to the complexity of the high-coverage mature biofilms.

In order to overcome the limits of the above methods, images were digitally processed with the semiautomated plugin of ImageJ/Fiji, termed OrientationJ, according to a workflow that did not require preliminary binarization and that selected the local structure tensor, that is, a matrix derived from the gradient of the image, to effectively investigate the local characteristics of the distribution of interest.

First of all, the tests on the bare substrate were considered to be the control. Furthermore, as the observed morphological pattern and growth evolution of the biofilm formations were ascertained to be independent of the particular substrate, the key role is played by the mutant and, definitively, the mutants act on growth in a specific manner.

The different social behaviors of the bacterial colonies, determined in this case by the mutations, were associated with a growth pathway that was disclosed and characterized by means of computational imaging methods and statistics approaches. The occurrence of local preferred directions and the increase in the local coherency pointed out in the real images seem to be systematically related to increasing coverage. The comparison of our findings with the literature enforced the consistency of these observations as well as pointed out relevant differences. In this respect, unlike the literature reports on the differential growth of *Vibrio cholerae* biofilms pointed out through simulations and the continuous modelling of low-coverage (thousands of cells) samples, our analysis were applied to mature biofilm formations, representing complex distributions prohibitive to being modelled.

Biofilms and the closely related phenomenon of QS are of fundamental importance in the bacterial and medical fields, as they correlate with genetic and adaptive resistance to antibiotics but also because they characterize the collective behavior of bacteria [2,3,4]. From a more specific biological point of view, it can be said that QS, one of the key targets for the development of new therapies, in the mutants can inhibit cell signaling circuits rather than having a bactericidal action. This type of approach has certain advantages, including not killing the bacteria and, therefore, avoiding the selective pressure that induces the emergence of resistant bacteria [60,61].

Finally, the developed approach is not only general in its implementation but also seems promising in the perspective of its application to other bacterial strains. In this respect, further research and tests are needed, and this will be the subject of future studies. For instance, the developed approach may be useful for studying various biofilm-forming pathogens, such as *V. cholerae* or Gram-negative (*Pseudomonas aeruginosa* [62]) and Gram-positive (*Acinetobacter baumannii* [63] or *Staphylococcus aureus* [64]) pathogens. In this regard, some data suggest that certain drugs developed for other purposes may influence QS. For example, metformin is a drug for the treatment of type II diabetes mellitus, but it has also been proposed as a QS inhibitor and virulence modulator that could be useful in the treatment of *P. aeruginosa* [65]. In this regard, the presented procedure could also be considered for the development of a highly efficient screening method for old and new drugs or for innovative materials which influence the modulation of QS and the inhibition of biofilm formations.

## Figures and Tables

**Table 1:** Measurements of the biomass (CFU/mL) for each strain, grown on the HA substrate, and of the bioluminescence signal.

STRAIN	CFU/mL	Bioluminescence
BB120	(1.0 ± 0.1) × 10^6^	(4.53 ± 0.45) × 10^8^
KM387	(1.2 ± 0.3) × 10^6^	(1.55 ± 0.10) × 10^6^
JAF633	(1.6 ± 0.5) × 10^6^	(1.22 ± 0.24) × 10^7^
JMH603	(2.7 ± 0.4) × 10^6^	(1.73 ± 0.44) × 10^8^

**Table 2:** Results of the local thresholding with the Niblack algorithm: radius of the local thresholding domain (R_Niblack_); number of objects in the segmented image (counts); total area of the segmented objects (A); change in the coverage of the segmented image with respect to the segmented image of BB120 (relative cvg); and the Voronoi regularity index (VRI) (*See the text for more detailed explanations.*).

STRAIN	*R_Niblack_*	Count	A (mm^2^)	Relative cvg (%)	VRI
BB120	150	4549	64.55	1%	0.116
KM387	200	7352	66.07	+2.36%	0.218
JAF633	70	6909	67.34	+4.33%	0.243
JMH603	400	3069	74.63	+15.61%	0.139

## Data Availability

The data presented in this study are available on request from the corresponding authors with specific restrictions related to their reuse and discussion in further publications.

## References

[1] Costerton J.W., Geesey G., Cheng K.J. (1978). How Bacteria Stick. Sci. Am..

[2] Flemming H.-C., Wingender J., Szewzyk U., Steinberg P., Rice S.A., Kjelleberg S. (2016). Biofilms: An emergent form of bacterial life. Nat. Rev. Microbiol..

[3] Flemming H.-C., Wingender J. (2010). The biofilm matrix. Nat. Rev. Microbiol..

[4] Yan J., Bassler B.L. (2019). Surviving as a Community: Antibiotic Tolerance and Persistence in Bacterial Biofilms. Cell Host Microbe.

[5] Echeverria C., Torres M.D.T., Fernández-García M., de la Fuente-Nunez C., Muñoz-Bonilla A. (2020). Physical methods for controlling bacterial colonization on polymer surfaces. Biotechnol. Adv..

[6] Jefferson K.K. (2004). What drives bacteria to produce a biofilm?. FEMS Microbiol. Lett..

[7] Sun L.-D., Dong H., Zhang P.-Z., Yan C.-H. (2015). Upconversion of Rare Earth Nanomaterials. Annu. Rev. Phys. Chem..

[8] Carniello V., Peterson B.W., van der Mei H.C., Busscher H.J. (2018). Physico-chemistry from initial bacterial adhesion to surface-programmed biofilm growth. Adv. Colloid Interface Sci..

[9] Song F., Koo H., Ren D. (2015). Effects of Material Properties on Bacterial Adhesion and Biofilm Formation. J. Dent. Res..

[10] Cozorici D., Măciucă R.-A., Stancu C., Tihăuan B.-M., Uță R.B., Codrea C.I., Matache R., Pop C.-E., Wolff R., Fendrihan S. (2022). Microbial Contamination and Survival Rate on Different Types of Banknotes. Int. J. Environ. Res. Public Health.

[11] Zhang C., Li B., Huang X., Ni Y., Feng X.-Q. (2016). Morphomechanics of bacterial biofilms undergoing anisotropic differential growth. Appl. Phys. Lett..

[12] Yildiz F., Visick K. (2009). Vibrio biofilms: So much the same yet so different. Trends Microbiol..

[13] You Z., Pearce D.J.G., Sengupta A., Giomi L. (2018). Geometry and Mechanics of Microdomains in Growing Bacterial Colonies. Phys. Rev. X.

[14] Nijjer J., Li C., Zhang Q., Lu H., Zhang S., Yan J. (2021). Mechanical forces drive a reorientation cascade leading to biofilm self-patterning. Nat. Commun..

[15] Yan J., Sharo A.G., Stone H.A., Wingreen N.S., Bassler B.L. (2016). Vibrio cholerae biofilm growth program and architecture revealed by single-cell live imaging. Proc. Natl. Acad. Sci. USA.

[16] Dell’Arciprete D., Blow M.L., Brown A.T., Farrell F.D.C., Lintuvuori J.S., McVey A.F., Marenduzzo D., Poon W.C.K. (2018). A growing bacterial colony in two dimensions as an active nematic. Nat. Commun..

[17] Porter M., Davidson F.A., MacPhee C.E., Stanley-Wall N.R. (2022). Systematic microscopical analysis reveals obligate synergy between extracellular matrix components during Bacillus subtilis colony biofilm development. Biofilm.

[18] Jiang S., Huang X., Zhang C., Cai Z., Zou T. (2015). Morphological and proteomic analyses of the biofilms generated by Streptococcus mutans isolated from caries-active and caries-free adults. J. Dent. Sci..

[19] Nerenberg R. (2016). The membrane-biofilm reactor (MBfR) as a counter-diffusional biofilm process. Curr. Opin. Biotechnol..

[20] Hobley L., Harkins C., MacPhee C.E., Stanley-Wall N.R. (2015). Giving structure to the biofilm matrix: An overview of individual strategies and emerging common themes. FEMS Microbiol. Rev..

[21] Solano C., Echeverz M., Lasa I. (2014). Biofilm dispersion and quorum sensing. Curr. Opin. Microbiol..

[22] Henke Jennifer M., Bassler Bonnie L. (2004). Three Parallel Quorum-Sensing Systems Regulate Gene Expression in Vibrio harveyi. J. Bacteriol..

[23] Hoang H.T., Nguyen T.T.T., Do H.M., Nguyen T.K.N., Pham H.T. (2022). A novel finding of intra-genus inhibition of quorum sensing in Vibrio bacteria. Sci. Rep..

[24] Lorenz N., Shin J.Y., Jung K. (2017). Activity, Abundance, and Localization of Quorum Sensing Receptors in Vibrio harveyi. Front. Microbiol..

[25] Lin B., Wang Z., Malanoski A.P., O'Grady E.A., Wimpee C.F., Vuddhakul V., Alves N., Thompson F.L., Gomez-Gil B., Vora G.J. (2010). Comparative genomic analyses identify the Vibrio harveyi genome sequenced strains BAA-1116 and HY01 as Vibrio campbellii. Environ. Microbiol. Rep..

[26] Austin B., Zhang X.H. (2006). Vibrio harveyi: A significant pathogen of marine vertebrates and invertebrates. Lett. Appl. Microbiol..

[27] Liu J., Fu K., Wu C., Qin K., Li F., Zhou L. (2018). “In-Group” Communication in Marine Vibrio: A Review of N-Acyl Homoserine Lactones-Driven Quorum Sensing. Front. Cell. Infect. Microbiol..

[28] Cao J.G., Meighen E.A. (1989). Purification and Structural Identification of an Autoinducer for the Luminescence System of Vibrio harveyi *. J. Biol. Chem..

[29] Chen X., Schauder S., Potier N., Van Dorsselaer A., Pelczer I., Bassler B.L., Hughson F.M. (2002). Structural identification of a bacterial quorum-sensing signal containing boron. Nature.

[30] Ng W.-L., Perez L.J., Wei Y., Kraml C., Semmelhack M.F., Bassler B.L. (2011). Signal production and detection specificity in Vibrio CqsA/CqsS quorum-sensing systems. Mol. Microbiol..

[31] Anetzberger C., Pirch T., Jung K. (2009). Heterogeneity in quorum sensing-regulated bioluminescence of Vibrio harveyi. Mol. Microbiol..

[32] Jeckel H., Drescher K. (2021). Advances and opportunities in image analysis of bacterial cells and communities. FEMS Microbiol. Rev..

[33] Abràmoff M.D., Magalhães P., Ram S.J. (2004). Image Processing with Image. J. Biophotonics Int..

[34] Püspöki Z., Storath M., Sage D., Unser M., De Vos W.H., Munck S., Timmermans J.-P. (2016). Transforms and Operators for Directional Bioimage Analysis: A Survey. Focus on Bio-Image Informatics.

[35] Moline M.A., Blackwell S.M., Case J.F., Haddock S.H.D., Herren C.M., Orrico C.M., Terrill E. (2009). Bioluminescence to reveal structure and interaction of coastal planktonic communities. Deep Sea Res. Part II Top. Stud. Oceanogr..

[36] Schneider C.A., Rasband W.S., Eliceiri K.W. (2012). NIH Image to ImageJ: 25 years of image analysis. Nat. Methods.

[37] Aurenhammer F. (1991). Voronoi Diagrams—A Survey of a Fundamental Geometric Data Structure. ACM Comput. Surv..

[38] Clemons T.D., Bradshaw M., Toshniwal P., Chaudhari N., Stevenson A.W., Lynch J., Fear M.W., Wood F.M., Iyer K.S. (2018). Coherency image analysis to quantify collagen architecture: Implications in scar assessment. RSC Adv..

[39] Matlab. https://www.mathworks.com/help/matlab/ref/rand.html,.

[40] Hoffmann H. (2020). Violin Plot. https://it.mathworks.com/matlabcentral/fileexchange/45134-violin-plot.

[41] Dorn J. (2020). Violin Plots for Plotting Multiple Distributions. https://www.mathworks.com/matlabcentral/fileexchange/23661-violin-plots-for-plotting-multiple-distributions-distributionplot-m.

[42] Gonzalez R.C. (2017). Digital Image Processing.

[43] Horn H., Lackner S., Muffler K., Ulber R. (2014). Modeling of Biofilm Systems: A Review. Productive Biofilms.

[44] Takashimizu Y., Iiyoshi M. (2016). New parameter of roundness R: Circularity corrected by aspect ratio. Prog. Earth Planet. Sci..

[45] Winkle J.J., Igoshin O.A., Bennett M.R., Josić K., Ott W. (2017). Modeling mechanical interactions in growing populations of rod-shaped bacteria. Phys. Biol..

[46] Farrell F.D., Gralka M., Hallatschek O., Waclaw B. (2017). Mechanical interactions in bacterial colonies and the surfing probability of beneficial mutations. J. R. Soc. Interface.

[47] Acemel R.D., Govantes F., Cuetos A. (2018). Computer simulation study of early bacterial biofilm development. Sci. Rep..

[48] Cesaria M., Alfinito E., Arima V., Bianco M., Cataldo R. (2022). MEED: A novel robust contrast enhancement procedure yielding highly-convergent thresholding of biofilm images. Comput. Biol. Med..

[49] Silingardi F., Bonvicini F., Cassani M.C., Mazzaro R., Rubini K., Gentilomi G.A., Bigi A., Boanini E. (2022). Hydroxyapatite Decorated with Tungsten Oxide Nanoparticles: New Composite Materials against Bacterial Growth. J. Funct. Biomater..

[50] Li B., Xia X., Guo M., Jiang Y., Li Y., Zhang Z., Liu S., Li H., Liang C., Wang H. (2019). Biological and antibacterial properties of the micro-nanostructured hydroxyapatite/chitosan coating on titanium. Sci. Rep..

[51] Cheng X., Liu J., Li J., Zhou X., Wang L., Liu J., Xu X. (2017). Comparative effect of a stannous fluoride toothpaste and a sodium fluoride toothpaste on a multispecies biofilm. Arch. Oral Biol..

[52] Peeters E., Nelis H.J., Coenye T. (2008). Comparison of multiple methods for quantification of microbial biofilms grown in microtiter plates. J. Microbiol. Methods.

[53] Pare S., Kumar A., Singh G.K., Bajaj V. (2020). Image Segmentation Using Multilevel Thresholding: A Research Review. Iran. J. Sci. Technol. Trans. Electr. Eng..

[54] Sahoo P.K., Soltani S., Wong A.K.C. (1988). A survey of thresholding techniques. Comput. Vis. Graph. Image Proc..

[55] Niblack W. (1986). An Introduction to Digital Image Processing.

[56] Keeley P.W., Eglen S.J., Reese B.E. (2020). From random to regular: Variation in the patterning of retinal mosaics *. J. Comp. Neurol..

[57] Dobrin A. (2005). A Review of Properties and Variations of Voronoi Diagrams.

[58] Jahne B. (1993). Patio-Temporal Image Processing: Theory and Scientific Applications.

[59] Rezakhaniha R., Agianniotis A., Schrauwen J.T.C., Griffa A., Sage D., Bouten C.V.C., van de Vosse F.N., Unser M., Stergiopulos N. (2012). Experimental investigation of collagen waviness and orientation in the arterial adventitia using confocal laser scanning microscopy. Biomech. Model. Mechanobiol..

[60] Defoirdt T. (2018). Quorum-Sensing Systems as Targets for Antivirulence Therapy. Trends Microbiol..

[61] Rabin N., Zheng Y., Opoku-Temeng C., Du Y., Bonsu E., Sintim H.O. (2015). Biofilm formation mechanisms and targets for developing antibiofilm agents. Future Med. Chem..

[62] Sharma G., Rao S., Bansal A., Dang S., Gupta S., Gabrani R. (2014). Pseudomonas aeruginosa biofilm: Potential therapeutic targets. Biologicals.

[63] Saipriya K., Swathi C.H., Ratnakar K.S., Sritharan V. (2020). Quorum-sensing system in Acinetobacter baumannii: A potential target for new drug development. J. Appl. Microbiol..

[64] Moormeier D.E., Bayles K.W. (2017). Staphylococcus aureus biofilm: A complex developmental organism. Mol. Microbiol..

[65] Abbas H., Shaldam M. (2017). Repurposing metformin as a quorum sensing inhibitor in Pseudomonas aeruginosa. Afr. Health Sci..

